# Quality and Readability of Web-Based Information for Patients With Pancreatic Cysts: DISCERN and Readability Test Analysis

**DOI:** 10.2196/25602

**Published:** 2021-03-16

**Authors:** Sven P Oman, Himesh Zaver, Mark Waddle, Juan E Corral

**Affiliations:** 1 Division of Hospital Internal Medicine Mayo Clinic Jacksonville, FL United States; 2 Department of Internal Medicine Mayo Clinic Jacksonville, FL United States; 3 Department of Radiation Oncology Mayo Clinic Rochester, MN United States; 4 Division of Gastroenterology and Hepatology Presbyterian Hospital Albuquerque, NM United States

**Keywords:** internet, pancreatic cyst, health literacy, patient education, pancreas, information seeking

## Abstract

**Background:**

Pancreatic cysts are a complex medical problem with several treatment options. Patients use web-based health information to understand their conditions and to guide treatment choices.

**Objective:**

The goal of this study was to describe the quality and readability of publicly available web-based information on pancreatic cysts and to compare this information across website affiliations.

**Methods:**

A Google search for “pancreatic cysts” was performed and the first 30 websites were evaluated. Website affiliations were classified as academic, media, nonprofit, government, or not disclosed. Information describing cancer risk was recorded. The DISCERN instrument measured the quality of content regarding treatment choices. Four standardized tests were used to measure readability.

**Results:**

Twenty-one websites were included. The majority of the websites (20/21, 95%) described the cancer risk associated with pancreatic cysts. Nearly half of the websites were written by an academic hospital or organization. The average DISCERN score for all websites was 40.4 (range 26-65.5, maximum 80). Websites received low scores due to lack of references, failure to describe the risks of treatment, or lack of details on how treatment choices affect quality of life. The average readability score was 14.74 (range 5.76-23.85, maximum 19+), indicating a college reading level. There were no significant differences across website affiliation groups.

**Conclusions:**

Web-based information for patients with pancreatic cysts is of moderate quality and is written above the reading level of most Americans. Gastroenterological, cancer treatment organizations, and physicians should advocate for improving the available information by providing cancer risk stratification, treatment impact on quality of life, references, and better readability.

## Introduction

The incidence of pancreatic cysts is increasing in developed countries owing to refined abdominal imaging and increasing use of imaging overall [1]. Although mostly benign, some types of pancreatic cysts have malignant potential and may transform into pancreatic cancer. Risk stratification is important to decide which patients should undergo surveillance with serial abdominal imaging (ie, magnetic resonance or endoscopic ultrasound) or which patients should be referred for surgical evaluation. A diagnosis of pancreatic cysts may, therefore, be a cause for concern in patients and may result in health information–seeking behavior. Approximately 70% of Americans use the internet to research health issues [2]. Although reviewing of web-based health information may empower patients to take a more active role in their treatment and improve their relationship, communication, and satisfaction during consultation with their treating physician, it may also introduce cyberchondria, and some websites have information that is of poor quality, is difficult to read, and inconsistent with medical practice guidelines [3-8]. Some of these problems exist because there is no regulation or oversight of web-based health information, which may result in information that is incomplete, unsupported, outdated, biased, or inappropriate for the average reader. Since web-based health information influences patients’ perceived understanding of health issues and how they manage their health, this information must be accurate and readable [9]. Of all the available internet search engines, Google represents 80%-91% of the internet searches and web-based advertising worldwide, with more than 63,000 searches completed every second [10,11]. In this study, we aimed to describe the quality and readability of publicly available web-based information on pancreatic cysts by using the most popular search engine in the world. The secondary objective was to compare the quality and readability among different website affiliations.

## Methods

A Google website search was performed from November 1, 2019 to December 31, 2019. Two reviewers (SO and HZ) independently rated the first 30 websites retrieved using the search term “pancreatic cysts.” Inclusion criteria were websites intended for the general public with more than 100 words. Websites with associated fees to access content, duplicate websites, publicly modifiable websites, and those with most of the content in audio or video format were excluded. The affiliation of the website was verified using the WHOis.net database (Table 1). For comparison, the same search was performed using Bing and Yahoo search engines on January 8 and 9, 2021, respectively. The quality of the content was measured with the DISCERN instrument (Table 1) [12].

DISCERN was developed by an expert panel of researchers, clinicians, health journalists, and consumers and is funded by The British Library and the NHS Research and Development Program. The instrument is comprised of 16 questions designed for consumers and information providers to assess the quality of written information about available treatment options for any health issue. Each question uses a rating scale from 1 to 5 (1=definite no, 2-4=partially, 5=definite yes) to indicate whether the publication has met certain criteria. The use of DISCERN does not require specialist knowledge or expertise since it is used to judge the reliability of the sources of information and not the scientific quality or evidence. The DISCERN instrument consists of 3 sections. Section 1 addresses the reliability of the publication, specifically, whether the aims were clear, whether these aims were achieved, whether they were relevant, clear, and up-to-date sources of information, whether the information was balanced and unbiased, and whether it referred to any areas of uncertainty. Section 2 evaluates the specific information on the treatment choice(s) presented, specifically, whether they were fully described, whether the benefits and risks and consequences of withholding treatment were mentioned, how the treatment option(s) affect the quality of life, and whether shared decision making was supported. Section 3 rates the overall quality of the source of information (eg, low, moderate, or high). Raters are encouraged to use their independent judgment and their ratings from the proceeding questions, that is, if the majority of questions scored below 2, then the publication would receive low quality; scores in the mid-range would be rated as moderate; and scores mostly rating 4 or above would be rated as high.

Readability was analyzed using 4 standardized tests: Flesch-Kincaid Grade Level, Gunning Fog Index, Simple Measure of Gobbledygook (SMOG) Readability Formula, and Coleman-Liau Index (score range from 5=5th grade level of education to 19 or more=doctorate, Table 1). Scores from each test were averaged. These tests measure the approximate grade level of education needed to understand the written text. Higher scores correspond to a higher grade level of reading. The Flesch-Kincaid Grade Level evaluates word and sentence length; words with more syllables and sentences with more words are rated as more complex and receive a higher score. The Gunning Fog Index works similarly, using the number of words per sentence and the number of complex words (ie, words with 3 or more syllables) to calculate a score. SMOG produces a score based on the number of words with 3 or more syllables in 10 sentence samples. The Coleman-Liau Index assesses the number of characters in a word. The Readable ContentPro Software was used to calculate each of these scores [13].

The websites were grouped into 5 affiliation categories: nonprofit organization, academic, communication/media, government, and affiliation not disclosed. DISCERN scores from the 2 reviewers and the 4 readability tests were summarized as average and median values. Kruskal-Wallis tests compared the differences between the website groups. Interobserver agreement and κ statistic were calculated. Information regarding the cancer risk of pancreatic cysts was also recorded (Table 1).

**Table 1:** Measuring instruments used for health information websites.

Parameters, question instruments		Description
**Initial questions**		
	Affiliation	Nonprofit organization (.org), academic (.edu), communication/media, government (.gov), private/affiliation not disclosed (.com)
	Cancer risk explanation	Mentions risk for pancreatic cancer
**Quality of information**		
	DISCERN instrument	16 questions × 1-5 points each. Minimum score: 16 points; maximum score: 80 points
**Readability**		
	Flesch-Kincaid Grade Level	Minimum score: 5 points, maximum score: 19 points 5 points: 5th grade 6 points: 6th grade 7 points: 7th grade 12 points: 12th grade 13 points: University 1st year 14 points: University 2nd year 15-16 points: University 3rd-4th year 17-18 points: Master’s and professional degree 19+ points: Doctorate
	Gunning Fog Index	Same scoring as mentioned for Flesch-Kincaid Grade Level
	Simple Measure of Gobbledygook Readability Formula	Same scoring as mentioned for Flesch-Kincaid Grade Level
	Coleman-Liau Index	Same scoring as mentioned for Flesch-Kincaid Grade Level

## Results

Of the 30 websites examined, 21 met the inclusion criteria. Five of the 21 (23%) were written by nonprofit organizations, 10 (45%) by an academic hospital or organization, 5 (23%) by communication/media websites, and 1 (5%) by an organization without disclosed affiliation. No government websites (.gov) were identified in our sample. Three of the 5 websites published by nonprofit organizations were written by physicians. The Bing and Yahoo searches yielded similar search results as Google. For all 3 search engines, Mayo Clinic’s website on pancreatic cysts was the first appearing search result. Healthline, Memorial Sloan Kettering Cancer Center, The National Pancreas Foundation, and MedicineNet were found in the top 10 websites for all 3 search engines. Hopkins Medicine, Columbia Surgery, and Harvard were common in the top 30 websites for all 3 search engines as well. Pancreatic cancer risk was explained in 20 of the 21 (95%) websites (Table 2).

**Table 2:** Quality scores for the websites describing pancreatic cysts (DISCERN questionnaire, 1-5 points for each question).

Order^a^	Cancer risk^b^	Is the publication reliable? Questions #1-8 Average (1-5 points)^c^	Quality of information on treatment choices, Questions #9-15 Average (1-5 points)^d^	Overall quality, Q#16 (1-5 points)^e^	Total score (Q#1-16)^f^	Reference
1st	Yes	3.3	2.1	3.5	45.5	[14]
2nd	Yes	2.3	3.2	3.5	44.5	[15]
3rd	Yes	2.6	2.0	3	37.5	[16]
5th	Yes	2.9	1.8	2.5	38.0	[17]
6th	Yes	1.7	2.8	2	35.0	[18]
7th	Yes	2.1	1.9	2.5	32.5	[19]
8th	Yes	2.6	2.0	2.5	37.0	[20]
9th	Yes	2.4	2.5	3.5	40.0	[21]
10th	Yes	2.5	2.3	2.5	38.5	[22]
11th	Yes	3.6	1.1	1.5	38.0	[23]
12th	Yes	2.6	3.2	3	46.0	[24]
14th	Yes	2.1	3.2	3.5	42.5	[25]
15th	Yes	2.0	2.4	2.5	35.0	[26]
16th	Yes	4.1	4.0	4.5	65.5	[27]
17th	Yes	3.5	4.4	4.5	63.0	[28]
18th	Yes	2.2	2.0	2.5	34.0	[29]
20th	Yes	2.1	1.8	2	31.0	[30]
22nd	Yes	3.1	2.0	2.5	41.0	[31]
24th	Yes	3.3	2.4	2	45.0	[32]
29th	Yes	2.0	1.9	2.5	32.0	[33]
30th	No	1.6	1.6	2	26.0	[34]

^a^Order of appearance. Seven websites did not meet the inclusion criteria: 4th (requires payment); 13th, 23rd, and 27th (not intended for the general public); and 19th, 21st, and 25th (require payment and not intended for the general public).

^b^Did the website mention the risk of pancreatic cancer associated with pancreatic cysts?

^c^Average reliability of the publications=2.3.

^d^Average quality of the publications=2.4.

^e^Average overall quality=2.8.

^f^Average total score=40.4.

**Figure 1:**
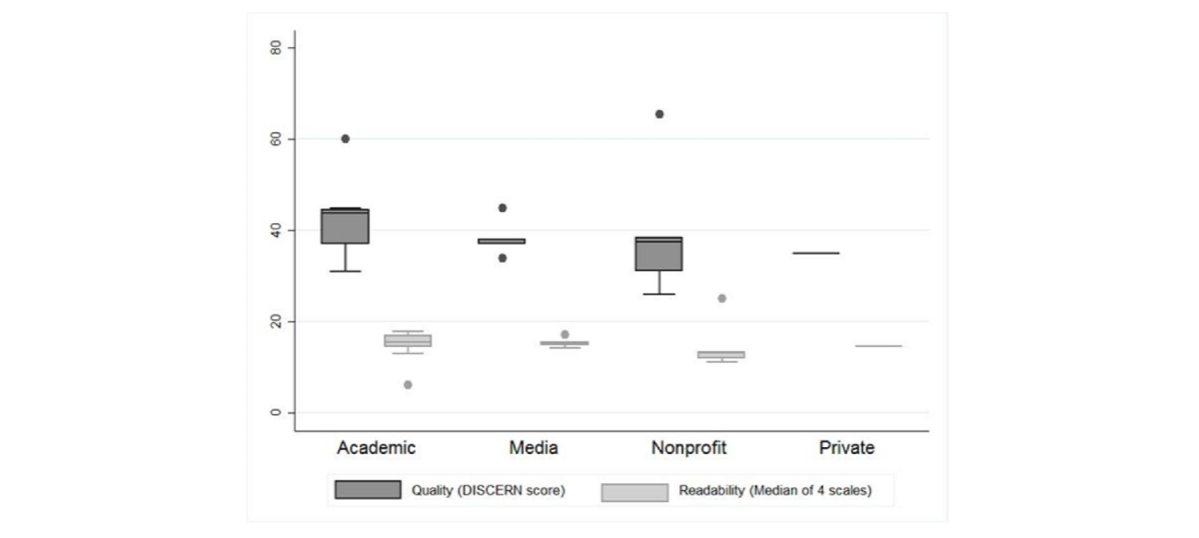
Box plot comparing website quality (DISCERN score, range 16-80 points) and readability (average of 4 instruments, range 5-19 points) by publisher affiliation. Lines represent upper and lower quartiles and dots are outliers.

The approach and depth to describe cancer risk stratification were diverse. One media website presented a new test for cyst fluid aspirate (monoclonal antibody Das-1) that may predict cancer risk [32]. One nonprofit website provided a complete diagnostic algorithm for patients to discuss with their health care provider, including cancer risk stratification and surgery [27]. One academic website provided a comprehensive 36-page review that included common signs and symptoms of growing cysts, types of pancreatic cysts, treatment options, an agenda for future appointments, and additional references [28]. The average total DISCERN score for all the websites was 40.4 (range 26-65.5, Table 2). The questions that, on average, received the lowest ratings on DISCERN were question #4: “Is it clear what sources of information were used to compile the publication?” (average score 2.14, range 1-5); question #11: “Does it describe the risks of each treatment?” (average score 1.93, range 1-4.5); and question #13: “Does it describe how the treatment choices affect the overall quality of life?” (average score 1.62, range 1-4.5). The median DISCERN score for nonprofit organizations was 33.5 (range 25-74), that for academic hospital or organization was 41 (27-65), that for communication/media was 38 (24-58), and that for an organization without disclosed affiliation was 35 (31-39) (Figure 1). The average total readability score for all the websites was 14.74 (range 5.76-23.9, Table 3).

The median (IQR) readability score for nonprofit organizations was 13.01 (8.64-31.76), that for academic hospital or organization was 15.59 (1.54-20.55), that for communication/media was 15.40 (11.5-19.31), and that for an organization without disclosed affiliation was 14.70 (12.86-16.86) (Figure 1). Scores were similar among website affiliations (DISCERN *P*=.90 and readability *P*=.80; Figure 1). Interobserver agreement was adequate (76.2%) but κ was poor (κ= –0.08).

**Table 3:** Readability scores for websites describing pancreatic cysts.

Order^a^	Affiliation	Average readability^b^	Reference
1st	Academic	14.26	[14]
2nd	Academic	16.00	[15]
3rd	Nonprofit	23.85	[16]
5th	Media	14.83	[17]
6th	Private group	14.78	[18]
7th	Academic	12.43	[19]
8th	Media	15.30	[20]
9th	Academic	15.49	[21]
10th	Nonprofit	12.72	[22]
11st	Media	13.87	[23]
12nd	Academic	14.93	[24]
14th	Academic	17.40	[25]
15th	Academic	18.04	[26]
16th	Nonprofit	11.94	[27]
17th	Academic	5.76	[28]
18th	Media	17.30	[29]
20th	Nonprofit	10.70	[30]
22nd	Academic	17.50	[31]
24th	Media	15.04	[32]
29th	Academic	14.85	[33]
30th	Nonprofit	12.69	[34]

^a^Order of appearance. Seven websites did not meet the inclusion criteria: 4th (requires payment); 13th, 23rd, and 27th (not intended for the general public); and 19th, 21st, and 25th (require payment and not intended for the general public).

^b^Average readability of all websites=14.74.

## Discussion

This study is the first, to our knowledge, to evaluate the quality and readability of web-based information for pancreatic cysts. The findings of this study highlight a substantial gap in the quality and readability of web-based information for patients with pancreatic cysts. Quality was suboptimal due to incomplete descriptions of pancreatic cyst management, lack of clear sources of information, and incomplete descriptions of the risks associated with treatment or how treatment choices affect the overall quality of life. Few websites provided a complete description of the management options (ie, radiological surveillance vs surgical treatment). Similar limitations have been described for other health conditions such as pancreatic cancer and gynecological disorders [4,5,7,35,36]. Despite the overall lack of high-quality web-based health information, a few sources were excellent. The top 5 highest DISCERN-rated websites were The American Gastroenterological Association (DISCERN score 65.5), The University of Michigan Comprehensive Cancer Center, GI Oncology Program (DISCERN score 63), Columbia Surgery (DISCERN score 46), Mayo Clinic (DISCERN score 45.5), and MedicalXpress (DISCERN score 45). We found 2 websites, a publication by the American Gastroenterological Association and a publication by the University of Michigan, which had DISCERN scores greater than 60 [27,28]. Across different health conditions, these high-quality websites represent the minority of cases. Three studies comparing the educational materials for obstetric and pelvic diseases reported that only 5% (3/58) [5], 15% (8/54) [7], and 4% (1/24) [4] of the materials achieved DISCERN scores greater than 60. Encouragingly, in our review, high-quality websites appeared as earlier search hits (our 2 high scorers appeared as the 16th and 17th hits), suggesting that they are more recognizable sources of information for internet users. 

We report median readability scores appropriate for a college reading level (range 13-16 points). This is problematic for most readers since only 35% of American citizens complete an undergraduate college education and most Americans read at an elementary school level (Table 4) [37].

**Table 4:** American population according to reading level (N=328 million).

Reading level	US population (millions), n (%)
6th grade	323 (98.5)
7th grade	317 (96.6)
12th grade	287 (87.5)
University 1st year	190 (57.9)
University 2nd year	127 (38.7)
University 3rd to 4th year	95 (28.9)
Master’s and Professional degree	33 (10.1)
Doctorate	7 (2.1)

Studies have similarly found that the readability levels of web-based health information are above the reading levels of most Americans [4,5,7,35,36]. The American Medical Association, therefore, recommends a sixth-grade reading level for all patient-oriented educational materials [36]. However, text difficulty increases from medical jargon and an effort to maintain quality and accuracy of health information. The top 5 most readable websites were The University of Michigan Comprehensive Cancer Center, GI Oncology Program (average readability score 5.76), Pancreatic Cancer UK (average readability score 10.70), The American Gastroenterological Association (average readability score 11.94), Roswell Park Comprehensive Cancer Center (average readability score 12.43), and Virginia Mason (average readability score 12.69). We found only 1 academic website that was written at an adequate reading level [28]. Previous studies found that only 13% (3/24) [4], 5% (3/58) [5], and 0% (0/54) [7] of the websites evaluated were written at an appropriate reading level. Our findings support the paradox that increased quality and accuracy come with a tradeoff of challenging language, longer article length, and higher readability scores [36,38]. 

The second important finding of this study was similar quality and accuracy scores among different website affiliations. This homogeneity of low quality among different publishers is common in web-based materials describing other health care conditions [4,38,39]. Although no difference was discernible in this study, possibly due to smaller sample size and higher variability, some prior studies have shown important differences. Academic sites have previously been found to be of high accuracy but more difficult to read, while private sites tend to be easier to read with lower accuracy of information [40]. Media websites are both difficult to read and have the lowest accuracy scores [36]. Differences have also been linked to the internet domain used. Research shows that organization domains (ie, “.org”) are an indicator of more accurate information [41] while sites that list references and those without financial interests are also associated with higher quality [42,43].

Our findings demonstrate the importance of balancing high-quality information (higher DISCERN scores) with lower reading levels (readability scores closer to 6) in patient handouts, websites, and reading materials [36,44]. For example, the material produced by the University of Michigan provides many pictorial representations of anatomy, pathology, procedures, and surgery. Houts et al [45] found that visual aids increase comprehension of complex medical information, which increases the understanding of health information, particularly among less literate patients [46]. Another avenue is patient testimonials. Although this study excluded websites that were mostly in the audiovisual format, video narrative presentations of breast cancer treatment wherein patients relate their experiences to other patients increase engagement with the material, with study participants spending more time viewing the information compared to text [47].

The Health of the Net was developed in 1995 to address issues with limited web-based health information quality and lack of supporting evidence. It is overseen by the Health of the Net Foundation in collaboration with the World Health Organization and consists of 8 principles that websites should follow to achieve the Code of Conduct (Health of the Net code) certification. The certification and display of the Health of the Net code seal are intended to help consumers identify reliable websites [48]. Another possible method to improve web-based information is for clinicians writing this information to consider why patients seek web-based information in the first place. For patients with cancer, seeking web-based information typically occurs right after receiving a diagnosis and before starting treatment. Patients may feel they received insufficient information from their providers and may turn to the internet to “fill in the gaps.” Information-seeking behavior is also a coping mechanism by which patients convince themselves that all treatment options have been explored [49]. Treating physicians should be aware of these reasons and redirect patients to credible and appropriate sites for information.

There are some limitations in this study design that are important to consider. First, our results are subject to selection bias and confounding. An examination of the first 30 results from our Google search represents a very small fraction of the websites that contain information regarding pancreatic cysts (eg, a search of “pancreatic cysts” renders over 2 million results on Google). Further, internet search engines use complex algorithms based on geographical locations and previous searches performed. Despite this, most internet users read only the first few pages of the results and 2 separate search engines yielded common results; thus, our analysis is likely a valid representation of an initial search a patient may conduct [50]. Additionally, our search was limited to the English language and our results have limited external validity outside the United States. The small sample size precluded advanced statistical analysis comparing findings among different website affiliations. Other limitations include the DISCERN instrument, which requires an element of subjective analysis, and the fact that our raters were not blinded to website affiliation. Readability tests also do not measure understanding of the material nor do they account for reader motivation, prior knowledge and attitudes, and problems such as poor vision and illness as well as the role of active voice, personalization, and presentation of the information, including font, font size, and illustrations [51,52]. These limitations underline the importance of follow-up with health care providers to clear up any potential misunderstandings and secure additional imaging or treatment when needed. Our study also did not analyze “information completeness” as a variable. Critical information such as genetic implications, costs of surveillance, and treatment was not recorded in our study design.

In summary, our findings demonstrate a gap in the quality and readability of web-based health information regarding pancreatic cyst management. These websites require peer review to balance improved quality with writing closer to a sixth-grade reading level. Gastroenterology and leading cancer organizations should advocate for improving web-based information by calling for a complete description of cancer risk stratification and treatments, visible sources of information and references, and appropriate readability, regardless of the website affiliation. These improvements can help less literate patients understand the information, reduce stress and anxiety after a new diagnosis, and facilitate shared decision making with providers.

## Abbreviations

SMOG: Simple Measure of Gobbledygook
